# Identification of Amino Acids in HA and PB2 Critical for the Transmission of H5N1 Avian Influenza Viruses in a Mammalian Host

**DOI:** 10.1371/journal.ppat.1000709

**Published:** 2009-12-24

**Authors:** Yuwei Gao, Ying Zhang, Kyoko Shinya, Guohua Deng, Yongping Jiang, Zejun Li, Yuntao Guan, Guobin Tian, Yanbing Li, Jianzhong Shi, Liling Liu, Xianying Zeng, Zhigao Bu, Xianzhu Xia, Yoshihiro Kawaoka, Hualan Chen

**Affiliations:** 1 Animal Influenza Laboratory of the Ministry of Agriculture and National Key Laboratory of Veterinary Biotechnology, Harbin Veterinary Research Institute, Chinese Academy of Agricultural Sciences, Harbin, People's Republic of China; 2 The 11th Institute, Academy of Military Medical Sciences, Changchun, People's Republic of China; 3 The International Center for Medical Research and Treatment, Kobe University, Kobe, Japan; 4 Division of Virology, Department of Microbiology and Immunology; International Research Center for Infectious Diseases, Institute of Medical Science, University of Tokyo, Tokyo, Japan; 5 Department of Pathobiological Sciences, University of Wisconsin-Madison, Madison, Wisconsin, United States of America; North Carolina State University, United States of America

## Abstract

Since 2003, H5N1 influenza viruses have caused over 400 known cases of human infection with a mortality rate greater than 60%. Most of these cases resulted from direct contact with virus-contaminated poultry or poultry products. Although only limited human-to-human transmission has been reported to date, it is feared that efficient human-to-human transmission of H5N1 viruses has the potential to cause a pandemic of disastrous proportions. The genetic basis for H5N1 viral transmission among humans is largely unknown. In this study, we used guinea pigs as a mammalian model to study the transmission of six different H5N1 avian influenza viruses. We found that two viruses, A/duck/Guangxi/35/2001 (DKGX/35) and A/bar-headed goose/Qinghai/3/2005(BHGQH/05), were transmitted from inoculated animals to naïve contact animals. Our mutagenesis analysis revealed that the amino acid asparagine (Asn) at position 701 in the PB2 protein was a prerequisite for DKGX/35 transmission in guinea pigs. In addition, an amino acid change in the hemagglutinin (HA) protein (Thr160Ala), resulting in the loss of glycosylation at 158–160, was responsible for HA binding to sialylated glycans and was critical for H5N1 virus transmission in guinea pigs. These amino acids changes in PB2 and HA could serve as important molecular markers for assessing the pandemic potential of H5N1 field isolates.

## Author Summary

H5N1 influenza viruses have caused over 400 human infections in 15 countries and continue to circulate in poultry and wild birds. Most human infections resulted from direct contact with virus-contaminated poultry or poultry products. It would be disastrous if H5N1 viruses acquired the ability to efficiently transmit among humans, because the mortality rate may exceed 60%. However, the genetic basis for transmission of H5N1 influenza viruses is largely unknown. Here, we demonstrate that the amino acid residue at 701 of PB2 is a prerequisite for transmission of H5N1 viruses in a mammalian guinea pig model. Interestingly, we found that the absence of glycosylation at residues 158–160 of the HA gene is pivotal for the H5N1 virus to bind to human-like receptors and to transmit in a mammal host. These findings are important for assessing the pandemic potential of H5N1 field isolates.

## Introduction

The H5N1 avian influenza viruses (AIVs) have attracted extensive attention for their deadly impact on both animals and humans. The number of people who have been subclinically infected with H5N1 viruses is very limited [1], and H5N1 AIVs have a 60% fatality rate in humans (World Health Organization [WHO]; http://www.who.int). H5N1 AIVs first surfaced in China in 1996 [2], with chicken-lethal and avirulent strains being isolated from geese in Guangdong province [3]. In 1997, a reassortant H5N1 AIV that carried the HA gene from an A/goose/Guangdong/1/96-like virus caused an outbreak of disease in poultry in Hong Kong and crossed over into humans, resulting in 18 cases of infection with six deaths [4],[5]. In 2003 and 2004, H5N1 AIVs infected poultry and humans in numerous countries of southeastern Asia [6]. In 2005, several genotypes of H5N1 AIV caused outbreaks in wild migratory birds at Qinghai Lake in western China, and one genotype spread widely to different species across a wide geographic area that included Europe and Africa [7]. To date, H5N1 AIVs have caused disease in more than 60 countries (Office International des Epizooties [OIE]; http://www.oie.int), with cases of human infection being reported in 15 countries (World Health Organization [WHO]; http://www.who.int). Despite substantial efforts to control these outbreaks, H5N1 AIVs have continued to evolve and spread, perpetuating the fear of an influenza pandemic if these viruses acquire the ability to transmit efficiently among humans. Understanding the genetic determinants that control H5N1 AIV transmission in mammalian hosts will help protect public health.

The transmissibility of influenza viruses is determined by the virus, environmental factors, and host factors [8]–[11]. The viral traits governing transmission efficiency have not been well characterized. The affinity of the viral HA protein for sialic acid α-2,6 linked glycan (α-2,6 glycan) is necessary for transmission of the 1918 H1N1 influenza virus between ferrets [9]. The viral polymerase complex is also involved in determining viral host range, replication and pathogenicity [12]–[15] and plays a role in transmission [16],[17]. Cold and dry environmental conditions favor the transmission of human influenza virus in guinea pigs [10],[11].

Host factors also affect replication and transmission of influenza viruses. Under experimental conditions, AIVs do not replicate efficiently in humans [18], and human viruses do not replicate efficiently in ducks [19]. In one study, the human upper airway tract was reported to contain cells that predominantly express α-2,6 glycan [20], an environment that favors the replication and transmission of human influenza viruses, which preferentially bind to α-2,6 glycans. However, in another study, viruses with preferential binding to α-2,3 glycans were reported to bind to cells in the human upper respiratory tract [21]. The first human isolates in the 1957 and 1968 pandemics preferentially bound to α-2,6 glycans even though their HAs were derived from avian viruses, which preferentially recognize α-2,3 glycans [22]. These findings suggest that for viruses with HAs from AIVs to be efficiently transmitted among humans, they need to preferentially bind to α-2,6 glycans [23].

Ferrets and guinea pigs have been successfully used as models to evaluate the transmissibility of AIVs and other species of influenza viruses in mammalian hosts [8], [9], [11], [16], [17], [24]–[30]. H5N1 AIVs exhibit varying levels of replication and virulence in mammalian mouse and ferret models [2],[13],[31]. In this study, we used guinea pigs as a mammalian model to examine the replication and transmission of six H5N1 AIVs that exhibit different replication and virulence phenotypes in mice. We also explored the genetic requirements for H5N1 AIV transmission in this mammalian host.

## Results

### Replication of H5N1 AIVs in guinea pigs

The six H5N1 AIVs used in this study have previously been shown to be lethal for chickens [2],[13], but to differ in their replication and lethality in mice (Table 1). To investigate the replication of these viruses in guinea pigs, two animals were intranasally inoculated with 10^6^EID_50_ of virus and euthanized three days post-inoculation (p.i.). Nasal wash, trachea, lung, brain, kidney, spleen and colon were collected from each animal for virus titration in eggs. A/duck/Guangxi/22/01 (DKGX/22), which does not replicate in mice [2],[13], was detected in the lung of one guinea pig on day 3 p.i., but not in the nasal wash or trachea of either of the other two inoculated animals (Table 1). The other five viruses were detected in the nasal washes, tracheas and lungs of both animals inoculated. Virus was not detected in the brains, kidneys, spleens, or colons of any of the animals inoculated. We also infected two animals for each virus and observed them for two weeks for signs of pathogenicity. After two weeks p.i., all of the animals seroconverted except for one of the two guinea pigs infected with the DKGX/22 virus (Table 1). None of the animals showed disease signs during the observation period. These results indicate that replication of H5N1 viruses in guinea pigs is restricted to the respiratory system.

**Table 1:** Replication of H5N1 avian influenza viruses in guinea pigs.

Virus (abbreviation)	Replication and virulence in mice^a^					Replication in guinea pigs^b^			
	Virus titers in organs(log_10_EID_50_/ml)				MLD_50_ (log_10_EID_50_)	Mean virus titers (log_10_EID_50_/gram)			Seroconversion (positive/total)^e^
	Lung	Spleen	Kidney	Brain		Nasal wash^c^	Trachea	Lung	
A/duck/Guangxi/22/01 (DKGX/22)	−	−	−	−	>6.5	−	−	0.8(1/2)^d^	1/2
A/duck/Fujian/17/01 (DKFJ/17)	1.4±0.4	−	−	−	>6.5	2.2±0.3	0.6±0.2	2.1±0.5	2/2
A/duck/Shanghai/13/01 (DKSH/13)	2.7±1.4	−	−	−	5.0	1.9±1.6	0.8±0.0	3.5±0.2	2/2
A/duck/Guangxi/35/01 (DKGX/35)	5.1±2.2	1.3±0.8	+	+	1.5	2.8±0.0	0.8±0.0	3.5±0.0	2/2
A/duck/Guangdong/22/02 (DKGD/22)	3.2±1.0	−	−	−	4.8	2.2±0.7	1.3±0.0	2.1±0.9	2/2
A/Bar-headed goose/Qinghai/3/05 (BHGQH/3)	6.3±0.9	2.0±0.3	2.5±0.3	2.9±0.7	<0.5	3.0±1.7	1.4±0.2	2.8±0.4	2/2

a Data shown are summarized from previous reports [2],[7]. Six-week-old BALB/c mice were infected i.n. with 10^6^ EID_50_ of each virus in a 50-µl volume. Organs were collected on day 3 p.i., and clarified homogenates were titrated for virus infectivity in eggs at initial dilutions of 1∶10 (lung), 1∶2 (other tissues), or undiluted if negative at the lowest dilution. + and −, virus was detected or not detected, respectively, in the undiluted samples.

b Groups of four guinea pigs were slightly anesthetized and intranasally inoculated with 10^6^EID_50_ of test virus in a 300µl volume, 150 µl per nostril. Two animals from each group were euthanized on day 3 p.i. and samples, including nasal wash, trachea, lung, spleen, kidney, colon and brain, were collected for virus titration in eggs. The remaining two animals were observed for two weeks and sera were collected at the end of the observation period. Virus was not detected in the spleen, kidney, colon and brain of any animals inoculated with the six viruses, therefore, the data from these samples are not shown in the table. −, virus was not detected in the undiluted sample.

c Data shown are log_10_EID_50_/ml.

d Virus was only detected in one of the two animals inoculated.

e Seroconversion was confirmed by hemagglutination inhibition (HI) assay.

### Transmission of H5N1 AIVs in guinea pigs

**Figure 1:**
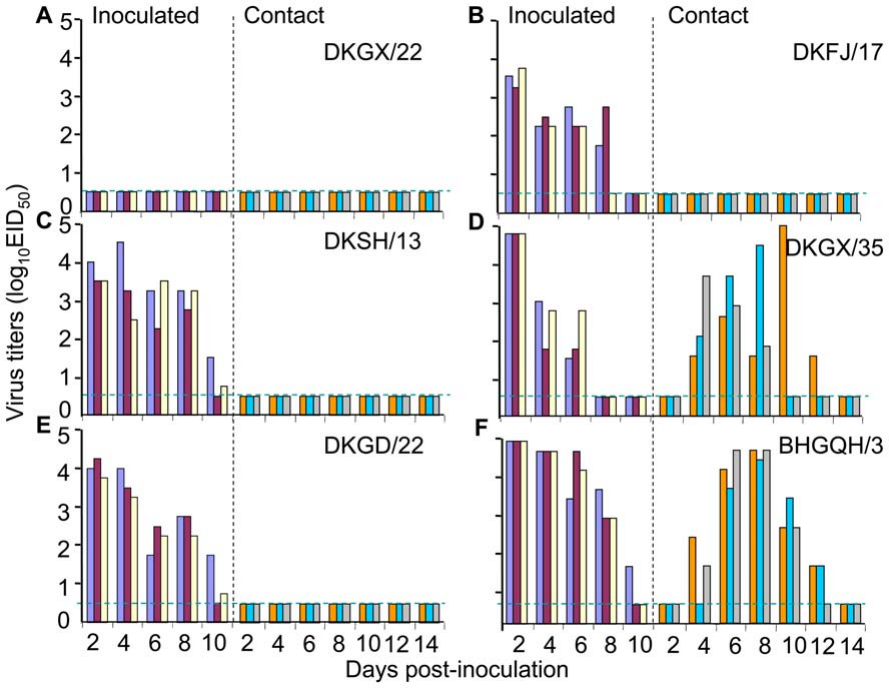
Transmisson of H5N1 avian influenza viruses in guinea pigs. Groups of three guinea pigs were inoculated i.n. with 10^6^EID_50_ of test virus and, 24 hours after the inoculation, three contact guinea pigs were placed in each cage. Nasal washes were collected every two days from all animals beginning 2 days p.i. for detection of virus shedding. (**A**) DKGX/22 virus; (**B**) DKGX/17 virus; (**C**) DKSH/13 virus; (**D**) DKGX/35 virus; (**E**) DKGD/22 virus; and (**F**) BHGQH/3 virus. Each color bar represents the virus titer from an individual animal. The dashed blue lines in these panels indicate the lower limit of detection.

Groups of three animals were inoculated with 10^6^ EID_50_ of each virus intranasally and three naïve animals were introduced into the same cage 24 h p.i. Evidence of transmission was based on the detection of virus in the nasal wash and on seroconversion at the end of the two-week observation period. As shown in Figure 1A, virus was not detected in the nasal washes of any inoculated or contact guinea pigs in the DKGX/22-inoculated group. In the DKFJ/17-, DKSH/13- and DKGD/22- inoculated groups, virus was detected in the nasal washes of all three inoculated guinea pigs between days 2–10 p.i., but not in any of the contact guinea pigs (Figure 1B,C,E). In the DKGX/35- and BHGQH/3-inoculated groups, virus was detected in the nasal washes of all three inoculated guinea pigs between days 2–6 or days 2–10 p.i., respectively and was also detected in the nasal washes of all three contact animals between days 4–12 p.i. (Figure 1D, F). Seroconversion occurred in all inoculated groups; however, in the DKGX/22-inoculated group, only two of the three animals seroconverted (Table 2). In the contact animal groups, seroconversion was only observed among animals placed with the DKGX/35- and BHGQH/3-inoculated animals. These results indicate that the transmissibility of H5N1 AIVs in guinea pigs varies among viral strains, and of the six test viruses, only DKGX/35 and BHGQH/3 transmit efficiently in this mammalian host.

**Table 2:** Seroconversion of the guinea pigs in our H5N1 avian influenza virus transmission studies.

Virus	Seroconversion: positive/total (HI titers)^a^	
	Inoculated	Contact
DKGX/22	1/3 (10)	0/3
DKFJ/17	3/3 (10,10,10)	0/3
DKSH/13	3/3 (40, 40,80)	0/3
DKGX/35	3/3(20,10,40)	3/3 (10, 10, 10)
DKGD/22	3/3 (80, 80, 80)	0/3
BHGQH/3	3/3 (10, 40,80)	3/3 (10, 10, 10)
35/PB2-701D	3/3 (10, 80, 80)	0/3
22/PB2-701N	3/3 (40, 160, 80)	0/3
35/HA-160T	3/3 (80, 160, 320)	0/3
35/HA-226L/228S	3/3 (40, 10, 10)	1/3 (10)
R-BHGQH/3	3/3 (40, 40, 20)	3/3 (20, 10, 10)
BHGQH/3-HA 160T	3/3 (40, 40,40)	0/3

a Sera were collected from guinea pigs on day 14 p.i. and treated overnight with *Vibrio cholerae* receptor-destroying enzyme. Seroconversion was confirmed by hemagglutination inhibition (HI) assay.

### PB2 amino acid 701N is required but not sufficient for H5N1 AIV transmission in guinea pigs

**Figure 2:**
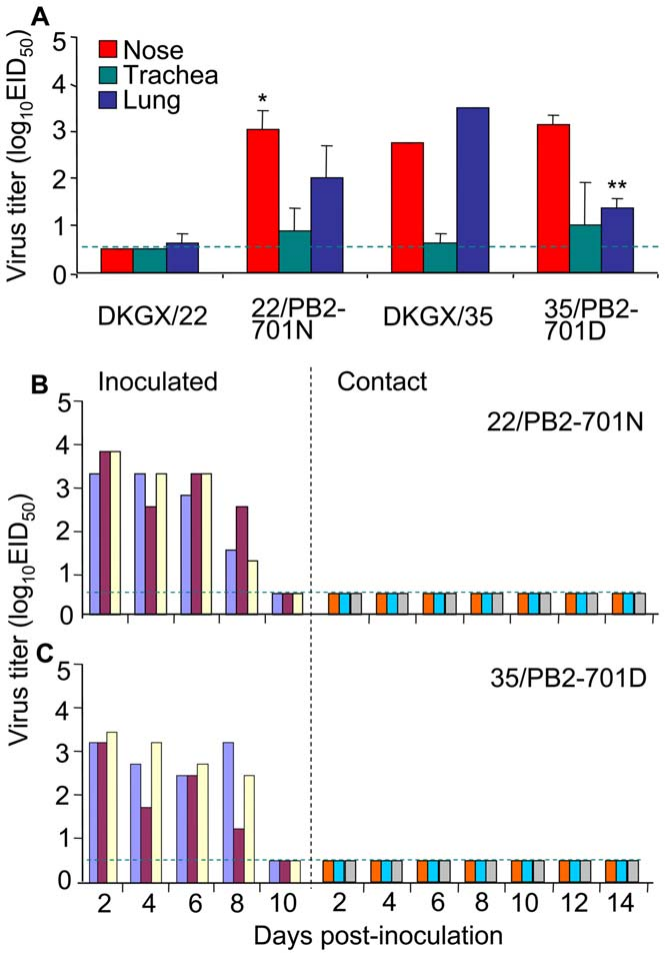
Replication and transmission of DKGX/35 PB2 mutants. (**A**) Groups of two guinea pigs were inoculated i.n. with 10^6^EID_50_ of test virus and then euthanized on day 3 p.i. Organs were collected for virus titration in eggs. (**B**) Transmission of the 22/PB2-701N virus in guinea pigs. (**C**) Transmission of the 35/PB2-701D virus in guinea pigs. The dashed blue lines in these panels indicate the lower limit of detection.

Of the six viruses tested, DKGX/22 and DKGX/35 have very similar genomes [2],[13]. Using DKGX/22 and DKGX/35, we previously determined that the amino acid at position 701 in the PB2 protein is important for H5N1 avian influenza virus replication in mice [13]. To determine whether this amino acid also contributes to the replication and transmission of H5N1 viruses in guinea pigs, we tested two mutants, DKGX/35-PB2-N701D (35/PB2-701D) and DKGX/22-PB2-D701N (22/PB2-701N) in guinea pigs. We found that when the D701N mutation was introduced in the PB2 protein (22/PB2-701N), the replication of the DKGX/22 virus dramatically increased. This mutant virus replicated in the nose, trachea, and lung of the guinea pigs at titer levels comparable to those observed in the DKGX/35-inoculated animals (Figure 2A). In the transmission experiment, 22/PB2-701N was detected in the noses of the inoculated guinea pigs from days 2–8 p.i., but no virus was detected in the contact animals (Figure 2B). The mutant 35/PB2-701D virus also replicated in the nose, trachea, and lung of the inoculated animals, although virus titers were appreciably lower than those observed in animals inoculated with wild-type DKGX/35 (Figure 2A). Transmission of 35/PB2-701D in guinea pigs was not detected (Figure 2C). These results indicate that PB2 701N is required, but not sufficient, for transmission of these duck H5N1 AIVs in guinea pigs, suggesting that other genes also contribute to this function.

### DKGX/35 H5N1 AIV binds to both α-2,3- and α-2,6-linked glycans

**Figure 3:**
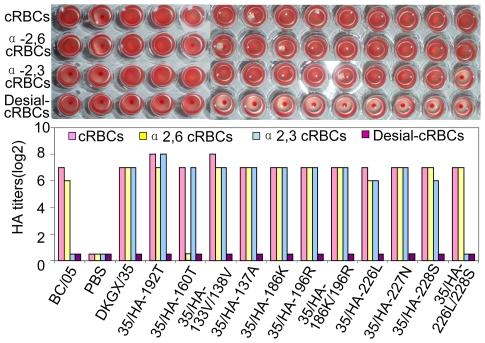
Hemagglutination assays of H5N1 influenza viruses using cRBCs with different treatments. The upper panel shows the hemagglutination by two HA units of each virus while the lower panel shows the HA titers of test viruses with 0.5% cRBCs treated as follows: cBRCs, untreated; Desial cRBCs, treated with VCNA; α-2,3 cRBC, VCNA treated and resialylated with α-2,3 glycans; α-2,6 cRBC,VCNA treated and resialylated with α-2,6 glycans.

HA receptor specificity plays an important role in the transmission of influenza viruses [9],[24],[26],[32]. The affinity of viral HA protein for α-2,6-glycan is required for the transmission of human influenza virus among ferrets [8],[9]. We examined the receptor-binding specificity of DKGX/35 by hemagglutination assays using resialyated cRBCs. DKGX/35 could bind to cRBCs resialylated with either α-2,3- or α-2,6-glycans, wheras the H1N1 human influenza virus BC/05 only bound to α-2,6 glycans (Figure 3).

**Figure 4:**
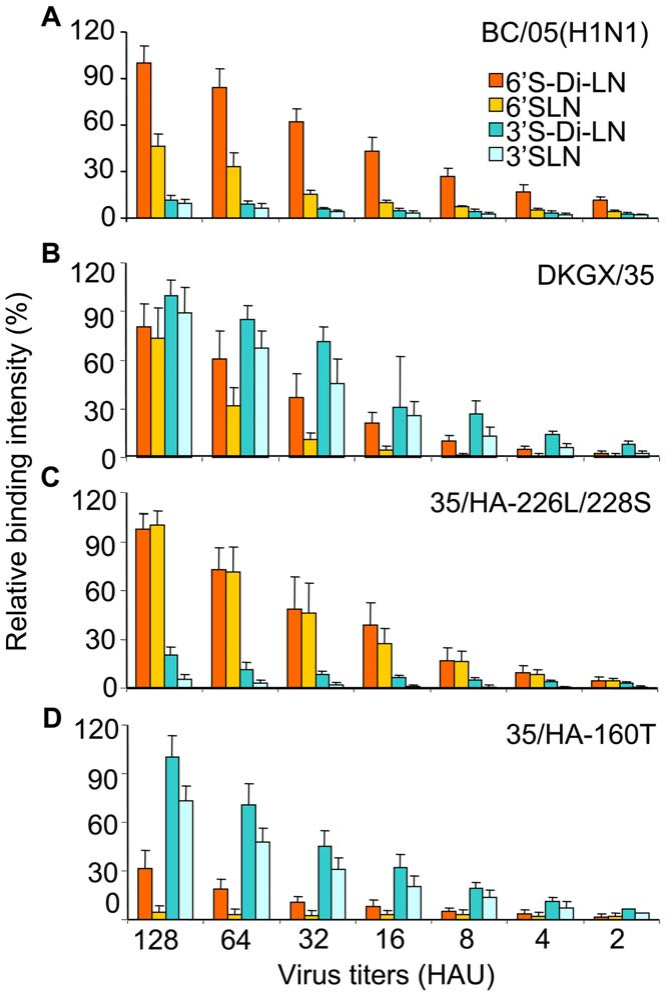
Glycan binding specificity of H1N1 and H5N1 viruses. (**A**) H1N1 human influenza BC/05 virus. (**B**) DKGX/35 virus. (**C**) 35/HA-226L/228S. (**D**) 35/HA-160T.

Chandrasekaran et al reported that the sialylated glycans in the α-2,3 and α-2,6 linkages have different topologies, and the HAs of H1N1 and H3N2 influenza viruses that have adapted to humans specifically bind to the long α-2,6 glycan topology, whereas the HA of H5N1 viruses, A/Vienam/1203/04 and A/Hong Kong/486/97, does not [33]. We tested the binding of the HAs of the DKGX/35 and BC/05 viruses with different glycans using a dose-dependent direct binding assay. As shown in Figure 4A, the human H1N1 virus BC/05 bound with high affinity to short and long α-2,6-linked glycans (6′SLN and 6′S-Di-LN) and showed minimal affinity for α-2,3-linked glycans (3′SLN and 3′S-Di-LN). DKGX/35 exhibited affinity for all four test glycans, although its affinity for the α-2-3-linked glycans was slightly higher than that for the α-2,6-linked glycans (Figure 4C). These results indicate that the HA of the avian H5N1 DKGX/35 virus binds to α-2,6-linked sialosides, including long α-2,6 glycans, as well as to α-2,3-linked sialosides.

### Mutation of the human influenza virus-like amino acids 226L and 228S of the DKGX/35 HA protein affects receptor binding specificity and decreases viral transmission in guinea pigs

Several amino acids within the H5N1 influenza virus HA gene are associated with receptor-binding specificity [34]–[39]. The DKGX/35 virus HA protein contains eight avian influenza virus-like amino acids (Table 3). To investigate how changing these eight amino acids to those found in human virus HAs affect H5N1 virus receptor-binding preference, we generated a series of mutants in the DKGX/35 virus background that contained one or two amino acid changes at these critical positions in the HA protein. We tested their receptor binding specificities by using hemagglutination assays. As shown in Figure 3, the mutants with the single amino acid changes of 137A, 186K, 196R, 226L, 227N and 228S retained the same receptor binding preference as the wild-type DKGX/35 virus. The same result was obtained with mutant viruses containing the double amino acid changes of 133V/138V and 186K/196R. However, the mutant containing the double amino acid change of 226L/228S (35/HA-226L/228S) bound to only α-2,6 glycan resialylated cRBCs and not α-2,3 glycan resialylated cRBCs. This lack of binding affinity to α-2,3 glycans was confirmed by using dose-dependent direct binding assays (Figure 4C). Together, these data indicate that the amino acids 226L and 228S of the HA protein are important for the receptor binding preference of the H5N1 influenza virus, as has been reported elsewhere [34],[37].

**Table 3:** Amino acids that may affect the receptor-binding specificity of the influenza virus HA gene.

Amino acid position: H3 number (H5 number)	Amino acid in virus			
	Most human influenza viruses	DKGX/35	BHGQH/3	Most avian influenza viruses
133(129)	Val (V)	Leu (L)	Leu (L)	Leu (L)
137(133)	Ala (A)	Ser (S)	Ser (S)	Ser (S)
138(134)	Val (V)	Ala (A)	Ala (A)	Ala (A)
160(156)	Ala (A)	Ala (A)	Ala (A)	Thr (T)
186(182)	Lys (K)	Asn (N)	Asn (N)	Asn (N)
192(188)	Ile (I)	Ile (I)	Thr (T)	Thr (T)
196(192)	Arg (R)	Gln (Q)	Gln (Q)	Gln (Q)
226(222)	Leu (L)	Gln (Q)	Gln (Q)	Gln (Q)
227(223)	Asn (N)	Ser (S)	Ser (S)	Ser (S)
228(224)	Ser (S)	Gly (G)	Gly (G)	Gly (G)

**Figure 5:**
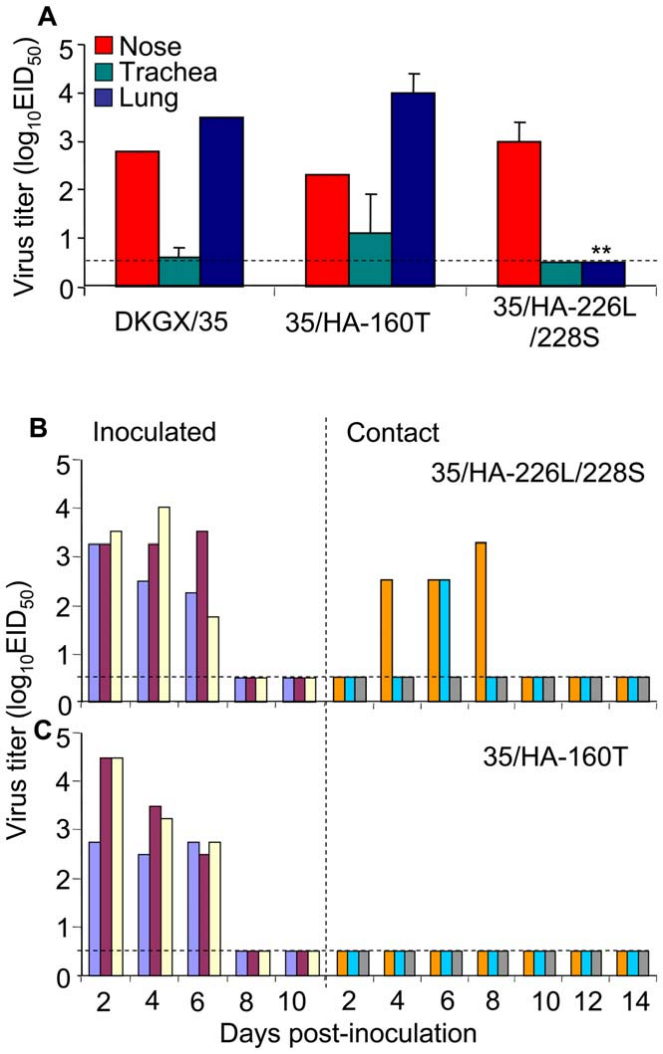
Replication and transmission of DKGX/35 HA mutants. (**A**) Groups of two guinea pigs were inoculated i.n. with 10^6^EID_50_ of test virus and euthanized on day 3 p.i. Organs were collected for virus titration in eggs. (**B**) Transmission of 35/HA-226L/228S in guinea pigs. (**C**) Transmission of 35/HA-160T in guinea pigs. The dashed blue lines in these panels indicate the lower limit of detection.

We then asked whether this change in receptor binding preference affected the replication and transmission of the mutant virus in guinea pigs. The 35/HA-226L/228S virus replicated as well as the wild-type DKGX/35 virus in the noses of guinea pigs, but did not replicate in their tracheas and lungs (Figure 5A). In the transmission study, the 35/HA-226L/228S virus was only detected in two of three contact animals (Figure 4C). These results indicate that the double amino acid mutations of Q226L and G228S in the HA protein abate the affinity of the DKGX/35 virus to bind α-2,3 glycans, and also slightly decrease its transmission among guinea pigs.

### Mutation of the avian influenza virus-like amino acid 160T of the DKGX/35 virus affects its receptor binding specificity and abolishes viral transmission in guinea pigs

As shown in Table 3, the DKGX/35 HA has two amino acids, 160A and 192I, that are conserved in most human influenza viruses. To investigate how these two amino acids affect the receptor-binding specificity of the H5N1 virus, we generated two mutant viruses in the DKGX/35 background, 35/HA-160T and 35/HA-192T, and analyzed their receptor binding preferences. 35/HA-160T bounds to α-2,3 glycan resialylated cRBCs but completely lost the ability to bind to α-2,6 glycan resialylated cRBCs, as determined by hemagglutination assays (Figure 3). The mutation I192T did not change the receptor binding preference of the DKGX/35 virus (Figure 3). The loss of receptor-binding affinity of 35/HA-160T to α-2,6 glycans was further confirmed by dose-dependent direct binding assays (Figure 4D).

We then tested the effect of the mutation A160T on the replication and transmission of DKGX/35 in guinea pigs. 35/HA-160T replicated as well as the wild-type DKGX/35 virus in the respiratory system of guinea pigs (Figure 4A), but was not detected in any of the contact animals (Figure 4B). These results indicate that the amino acid 160A of the HA protein is required for the H5N1 virus to bind the human α-2,6 sialic acid cellular receptors and for its transmission among guinea pigs.

**Figure 6:**
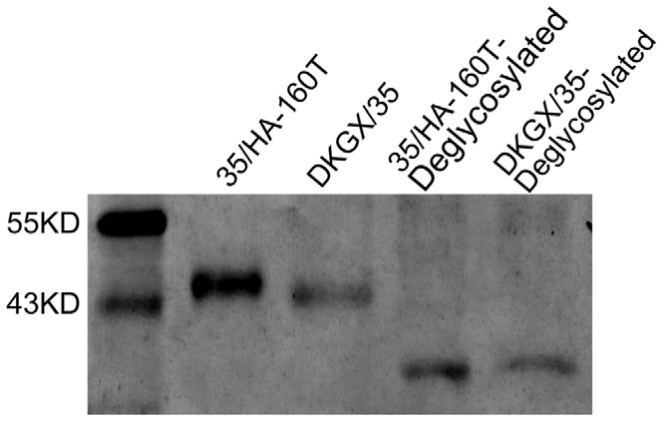
Western blot analyses of H5N1 avian influenza HA1 protein. Lysates of H5N1 viruses treated with or without PNGase F were incubated with chicken anti-H5N1 antiserum. Binding was visualized with 3,30-diaminobenzidine after incubation with peroxidase-conjugated secondary antibodies. The locations of marker proteins are indicated on the left.

The amino acid mutation of A to T at position 160 of the DKGX/35 HA protein formed a new potential N-linked glycosylation site -NST- at amino acid positions 158–160. To investigate whether this potential N-linked glycosylation site is indeed glycosylated, we performed Western blot analysis of the HA polypeptides of DKGX/35 and 35/HA-160T treated with or without PNGase F enzyme. As shown in Figure 6, the HA1 polypeptide of 35/HA-160T exhibited decreased mobility relative to that of DKGX/35 as a result of the single amino acid mutation of A160T, whereas when the HA1 polypeptides of these two viruses were deglycosylated with PNGase F, they showed similar mobility (Figure 6). These results suggest that the potential N-linked glycosylation site at amino acid positions 158–160 in the HA protein of the 35/HA-160T virus is glycosylated.

### The amino acid A160T mutation in the HA protein of the AIV BHGQH/3 altered its receptor-binding preference and abolished its transmission in guinea pigs

**Figure 7:**
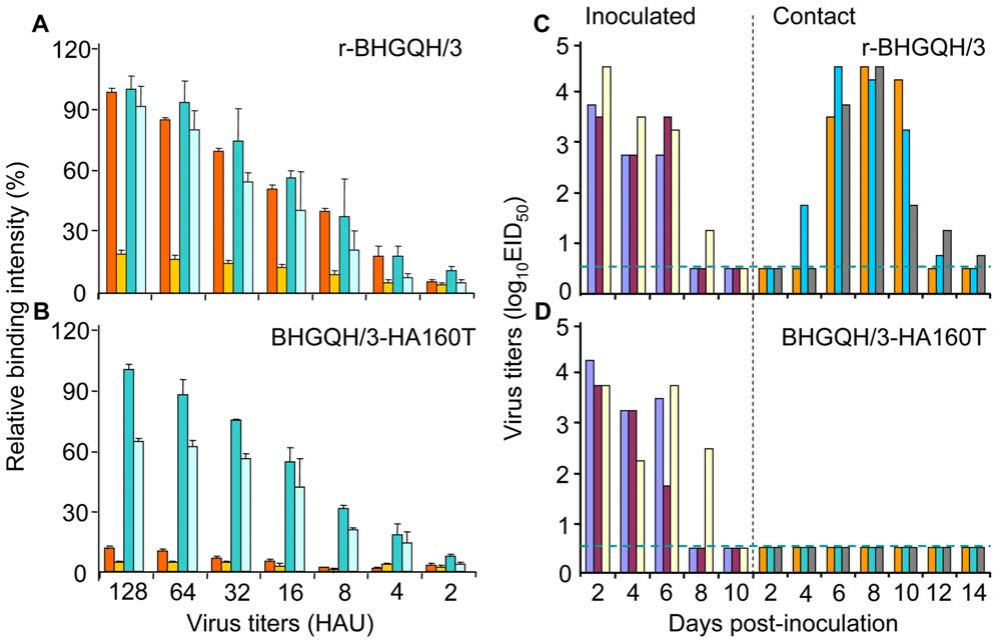
Receptor-binding preference and transmission of BHGQH/3 and its HA mutant. Receptor-binding preference of r-BHGQH/3 (**A**) and BHGQH/3-160T (**B**) were performed by dose-dependent direct binding assay as described in the text. (**C**) and (**D**) Transmisson of H5N1 duck viruses in guinea pigs. (C) r-BHGQH/3-inoculated group. (D) BHGQH/3-160T-inoculated group. The dashed blue lines in these panels indicate the lower limit of detection.

BHGQH/3 bears the amino acid A at the position 160 in its HA gene, as does DKGX/35 (Table 4). To test how the HA amino acid mutation A160T affects the receptor-binding and transmission properties of BHGQH/3, we introduced this mutation in the BHGQH/3 background by using reverse genetics. The rescued wild-type virus, r-BHGQH/3, bound to both α-2,3 and α-2,6 glycans (Figure 7A), whereas the mutant BHGQH/3-A160T bound to only α-2,3 glycans, as determined by dose-dependent direct binding assays (Figure 7B). These results were confirmed by use of hemagglutination assays (data not shown).

**Table 4:** Primers used to generate mutations in the HA gene of our H5N1 influenza viruses.

Mutation	Primer sequence (5′-3′)	
	Forward	Reverse
DKGX/35 I192T	CCTAATGATGCGGCAGAGCAGA**C**AAAGCTCTATC	GATAGAGCTTT**G**TCTGCTCTGCCGCATCATTAGG
DKGX/35 A160T	GGCTTATCAAAAAGAACAGTA**C**ATACCCAACAATAAAGAGG	CCTCTTTATTGTTGGGTAT**G**TACTGTTCTTTTTGATAAGCC
DKGX/35 S133V&A138V	CAATCATGAAGCCTCA**GT**AGGGGTGAGCTCAGTATGTCCATACC	GGTATGGACAT**AC**TGAGCTCACCCCT**A**CTGAGGCTTCATGATTG
DKGX/35 S137A	CATCAGGGGTGAGC**G**CAGCATGTCCATACCTG	CAGGTATGGACATGCTG**C**GCTCACCCCTGATG
DKGX/35 N186K	GGATTCACCATCCTAA**G**GATGCGGCAGAGCAGAT	ATCTGCTCTGCCGCATC**C**TTAGGATGGTGAATCC
DKGX/35 Q196R	GCAGAGCAGATAAAGCTCTATC**G**AAACCCAACCACC	GGTGGTTGGGTTTCGATAGAG**C**TTTATCTGCTCTGC
DKGX/35 Q226L	CCAAAGTAAACGGGC**T**AAGTGGAAGAATGGAGTTCTTC	GAAGAACTCCATTCTTCCACTT**A**GCCCGTTTACTTTGG
DKGX/35 S227N	CCAAAGTAAACGGGCAAA**A**TGGAAGAATGGAGTTCTTC	GAAGAACTCCATTCTTCCA**T**TTTGCCCGTTTACTTTGG
DKGX/35 G228S	CCAAAGTAAACGGGCAAAGT**A**G**T**AGAATGGAGTTCTTC	GAAGAACTCCATTCT**A**C**T**ACTTTGCCCGTTTACTTTGG
DKGX/35 Q226L&G228S	CCAAAGTAAACGGGC**T**AAGT**A**G**T**AGAATGGAGTTCTTC	GAAGAACTCCATTCTACTACTT**A**GCCCGTTT**A**CTTTGG
BHGQH/3 A160T	GGCTTATCAAAAAGAACAATA**C**ATACCCAACAATAAAGAG	CTCTTTATTGTTGGGTAT**G**TATTGTTCTTTTTGATAAGCC

The nucleotides that have been changed are underlined and in boldface.

We also compared the transmissibility of BHGQH/3-A160T in guinea pigs to that of the rescued r-BHGQH/3 virus. As shown in Figure 7C, the r-BHGQH/3 virus was detected in guinea pig nasal washes obtained between days 2–8 p.i. The virus was also detected in contact animals between days 4–14 p.i. BHGQH/3-A160T, however, was found in the nasal washes of only the inoculated animals, not the contact guinea pigs (Figure 7D). These results indicate that the amino acid mutation A160T in the HA protein of BHGQH/3 also abolishes the its binding affinity for α-2,3 glycans and its transmissibility in guinea pigs.

### Analysis of the receptor distribution in the respiratory system of guinea pigs

**Figure 8:**
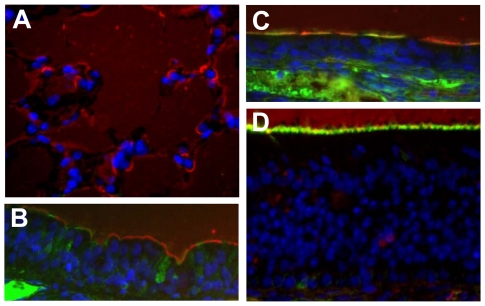
Receptor distribution in the respiratory system of guinea pigs. (**A**) Alveoli. Red staining indicates MAAII-binding α-2,3 glycan. (**B**) Tracheal mucosa of guinea pig. Red staining indicates the presence of α-2,3 glycan and green staining indicates the presence of α-2,6 glycan. (**C**) Nasal mucosa (respiratory region) of guinea pig. (**D**) Nasal mucosa (olfactory region) of guinea pig. Green staining indicates the presence of α-2,6 glycan.

To understand the underlying mechanism of the receptor binding specificities and replication phenotypes in guinea pigs of the viruses used in our experiments, we examined the receptor specificity of the respiratory tract of guinea pigs. The alveolar surface was covered by MAAII-binding α-2,3 glycan (Figure 8A, red), as was the surface of tracheal cells (Figure 8B, red), although some cells also expressed SNA-binding α-2,6 glycan (Figure 8B, green). The nasal respiratory region contained a mixture of cells expressing α-2,3 glycan and α-2,6 glycan (Figure 8C). In the nasal olfactory region, the cell surfaces were mostly covered by α-2,3 glycan (Figure 8D). The lack of α-2,6 glycans on the alveolar surface may explain the inability of the 35/HA-226L/228S virus to replicate in the lungs of guinea pigs (Figure 4A).

## Discussion

H5N1 AIVs have caused the deaths of more than half of the humans they have infected since 1997 and clearly represent a threat to public health (World Health Organization [WHO]; http:// www.who.int). Most human cases of infection resulted from direct exposure to H5N1 virus-infected poultry or poultry products. However, human-to-human transmission, albeit limited, has been detected [40],[41]. The effects of specific amino acid changes on the transmissibility of H5N1 highly pathogenic AIVs remain largely unexplored. Here, we evaluated the replication and transmission in guinea pigs of six H5N1 AIVs isolated in China between 2001 and 2005. We found that two of these viruses, DKGX/35 and BHGQH/03, not only replicated but also transmitted efficiently in guinea pigs. We demonstrated that the amino acid 701N in the PB2 protein is required for DKGX/35 transmission in guinea pigs, and that DKGX/35 and BHGQH/03 bind to both α-2,3 and α-2,6 glycans. The amino acid mutation A160T in the HA protein, which creates a new potential N-linked glycosylation site, abolishes the ability of viruses to bind α-2,6 glycans and to transmit in guinea pigs. Our results demonstrate that the PB2 and HA genes play important roles in the transmission of H5N1 influenza viruses in a mammalian host, and we are the first to report that the lack of glycosylation at amino acid positions 158–160 in HA is important for H5N1 AIVs to bind to the human receptor and to transmit in a mammalian host.

PB2, together with the viral proteins PB1 and PA, makes up the viral RNA polymerase. PB2 is an important determinant for the host range and virulence of influenza viruses [12]–[14],[42]. Two amino acids in PB2, 627K and 701N, have been found in H5N1 influenza viruses isolated from humans [43]. These amino acids are known to affect the replicative efficiency of H5N1 influenza A viruses in mice [12],[13]. Indeed, the amino acid 627K has been reported to favor the replication of H5N1 influenza virus in the upper respiratory tracts of mice and may be important for efficient person-to-person virus transmission [15]. Steel et al. [16] found that the amino acid residues 701N and 627K of PB2 increased the transmission of both human and avian influenza viruses in guinea pigs. In our study, we found that the amino acid mutation of N to D at position 701 of PB2 completely abolished the transmission of DKGX/35 virus among guinea pigs. The D to N mutation at position 701 of PB2 dramatically increased the replication of DKGX/22 in guinea pigs, but it did not confer transmissibility. Similarly, the mutant viruses 35/HA-160T and BHGQH/3-HA160T were unable to transmit in guinea pigs, even though both viruses contain either 701N or 627K in the PB2 protein. These results indicate that 701N or 627K in the PB2 protein are prerequisites, but not sufficient, for H5N1 virus transmission in a mammalian host.

The receptor binding specificity of HA has been implicated in the transmissibility of influenza viruses [9],[17]. Despite the ability to infect and cause severe disease in humans, most H5N1 viruses do not bind the α-2,6 glycan receptor with high affinity [34],[37],[39]. This low receptor-binding affinity is likely a major factor preventing H5N1 viruses from efficiently transmitting from person to person and causing a pandemic [44]. Although several reports have documented mutants of H5N1 influenza viruses that have modest affinity for α-2,6 glycans [34]–[39], it is unknown whether these mutations affect the transmission of the H5N1 influenza viruses in mammals. Here, we found that two H5N1 viruses, DKGX/35 and BHGQH/3, exhibited binding affinity for both α-2,3 and α-2,6 glycans and were able to transmit among guinea pigs. We demonstrated that the HA amino acid 160A plays a key role in the affinity of these two viruses for α-2,6 glycans. The single HA amino acid mutation of A160T abolished not only the binding affinity of DKGX/35 and BHGQH/3 to α-2,6 glycans, but also the transmission of these viruses among guinea pigs. It should be noted that viral transmission is a polygenic trait and that mutation of HA alone does not confer transmissibility to a virus.

Previous studies have reported that N-linked glycans close to the HA receptor binding domain can affect receptor-binding preference through steric hindrance or other mechanisms [45],[46]. The potential N-linked glycosylation site at HA amino acid positions 158–160 is commonly detected in H5N1 influenza viruses, but the majority of the clade 2.2 H5N1 isolates that were first isolated from wild birds in western China [7] and then in Europe, Africa and the Middle East, notably lack this potential N-linked glycosylation site [34]. Here, we demonstrated that the lack of this potential glycosylation site at HA amino acid positions 158–160 is critical for the H5N1 influenza viruses to bind α-2,6 glycans and to transmit in a mammalian host.

The HA amino acids at positions 226 and 228 play key roles in the receptor binding preference of influenza viruses [47],[48]. The HAs of human influenza viruses bear Leu at position 226 (226L) and Ser at position 228 (228S) and preferentially recognize α-2,6 glycans [48]. Although H5N1 influenza viruses have caused over 400 cases of human infection, the HA residues 226L and 228S have not been detected in any isolates, based on the available sequence information. Some naturally occurring H5N1 influenza mutants and lab-created H5N1 influenza viruses that contain mutations in the receptor-binding site of the HA protein, including the HA Q226L and G228S double mutant in the A/Vietnam/1203/2004 virus background, exhibit increased binding affinity for α-2,6 glycans, yet retain binding affinity for α-2,3 glycans [34],[37]. Here, we found that the mutant 35/HA-226L/228S, with the double HA amino acid mutation of Q226L and G228S in the DKGX/35 background, completely lost its ability to bind to α-2,3 glycans and only bound to α-2,6 glycans. It is likely that the HA amino acid 160A, which does not encode a potential glycosylation site at 158–160, also contributed to the inability of 35/HA-226L/228S to bind α-2,3 glycans.

Although the mutant virus 35/HA-226L/228S acquired human influenza virus-like receptor binding preference, its transmission in guinea pigs was slightly impaired relative to that of the wild-type DKGX/35 virus. Our receptor specificity analysis revealed that both α-2,3 and α-2,6 glycans were present in the nasal mucosa and the trachea mucosal surface, yet only α-2,3 glycans were detected in the alveoli of guinea pigs. This type of receptor distribution may favor the replication and transmission of an avian virus that can bind both α-2,3 and α-2,6 glycans, although human influenza viruses, which bind to only α-2,6 glycans, transmit well in guinea pigs [16]. Therefore, although the 35/HA-226L/228S virus cannot transmit efficiently in guinea pigs, it may be able to transmit easily in humans, which have a different receptor distribution than guinea pigs [20].

In summary, here, we demonstrated that the PB2 and HA proteins are important for the transmission of H5N1 influenza viruses in a mammalian host. We confirmed that the PB2 amino acid 701N is important for the transmission of H5N1 influenza virus in guinea pigs, and found, for the first time, that the T to A mutation at position 160, which results in the lack of an oligosaccharide side chain at 158–160 of HA, is critical for the H5N1 influenza viruses tested to bind to human-like receptors and to transmit among a mammalian host. The absence of a potential N-linked glycosylation site at HA amino acid positions 158–160 may serve as an important molecular marker for assessing the pandemic potential of H5N1 field isolates. Moreover, it is worrisome that clade 2.2 viruses bearing PB2 and HA mutations that permit transmission among mammalian hosts continue to circulate in wild birds and poultry across a wide geographic area. Clearly, there is a critical need for continued surveillance of poultry and regularly updated control measures.

## Materials and Methods

### Facility

Studies with highly pathogenic H5N1 avian influenza viruses were conducted in a biosecurity level 3+ laboratory approved by the Chinese Ministry of Agriculture. All animal studies were approved by the Review Board of Harbin Veterinary Research Institute, Chinese Academy of Agricultural Sciences.

### Viruses and cells

Human embryonic kidney cells (293T) were maintained in Dulbecco's modified Eagle's medium supplemented with 10% fetal bovine serum and incubated at 37°C and 5% CO_2_. The six H5N1 AIVs used in this study (Table 1) were isolated from ducks in southern China and wild birds at Qinghai Lake in northwest China as described previously [2],[7]. The construction of two PB2 mutant viruses, 22/PB2-701N and 35/PB2-701D, was reported previously [13]. An H1N1 virus isolated from humans in 2005, A/Baicheng/1/05(BC/05), was kindly provided by the Jilin Disease Control and Prevention Centers in China. The viruses were propagated in 10-day-old specific-pathogen-free (SPF) embryonated chicken eggs and were stored at −70°C.

### Guinea pig studies

Hartley strain female guinea pigs weighing 300–350 g and serologically negative for influenza virus were used in these studies. Ketamine (20 mg/kg) and xylazine (1 mg/kg) were used to anesthetize animals by intramuscular injection.

To investigate the replication of H5N1 AIVs, groups of four animals were anesthetized and inoculated intranasally (i.n.) with 10^6^ EID_50_ of test virus in a 300 µl volume (150 µl per nostril). Two animals from each group were euthanized on day 3 post inoculation (p.i) and nasal washes, tracheas, lungs, brains, kidneys, spleens, and colons were collected for virus titration in eggs. The remaining two animals were observed for two weeks for signs of disease and death.

For the contact transmission studies, groups of three animals were inoculated i.n. with 10^6^ EID_50_ of test virus and housed in a cage placed inside an isolator. Three naïve animals were introduced into the same cage 24 h later. Nasal washes were collected at 2 day intervals, beginning on day 2 p.i. (1 day post contact) and titrated in eggs. To prevent inadvertent physical transmission of virus by the investigators, the contact guinea pigs were always handled first, and gloves, implements, and napkins on the work surface were changed between animals. The ambient conditions for these studies were set as 20–22°C and 30%–40% relative humidity. The airflow in the isolator was horizontal with a speed of 0.1 m/s.

### Receptor-binding analysis using hemagglutination assays

Hemagglutination assays using resialyated chicken red blood cells (cRBCs) were performed as described previously [49],[50] with minor modifications. cRBCs were enzymatically desialyated with *Vibrio cholerae* neuraminidase (VCNA; Roche, www.roche.com), followed by resialylation using either α2–6-(N)-sialyltransferase or α2–3-(N)-sialyltransferase (Calbiochem, www.calbiochem.com) and CMP-sialic acid (Sigma, www.sigmaaldrich.com).

### Dose-dependent direct binding to different glycans of H5N1 avian influenza viruses

Analysis of the receptor specificity of influenza virus was performed by using a direct solid-phase assay [33]. Briefly, a streptavidin-coated, high-binding capacity 96-well plate (Pierce, www.piercenet.com) was rinsed with PBS and 50µl of a 2.4 mM solution of biotinylated glycans in PBS was added to each well and incubated overnight at 4°C. Two α-2,6 glycans (6′SLN Neu5Aca2-6Galb1-4GlcNAcb-SpNH-LC-LC-Biotin, 6′S-Di-LN Neu5Aca2-6[Galb1-4GlcNAcb1-3]_2_b-SpNH-LC-LC-Biotin) and two α-2,3 glycans (3′SLN: Neu5Aca2-3Galb1-4GlcNAcb-SpNH-LC-LC-Biotin, 3′S-Di-LN Neu5Aca2-3[Galb1-4GlcNAcb1-3]_2_b-SpNH-LC-LC-Biotin) were tested upon being kindly provided by the Consortium for Functional Glycomics (Scripps Research Institute, Department of Molecular Biology, La Jolla, CA). The plate was subsequently washed with cold PBS to remove any excess glycans. Virus was inactivated by adding 0.1% (v/v) β-propiolactone for 3 days at 4°C. Virus binding to the glycan-coated wells was performed by adding serially diluted virus in PBS containing 1% bovine serum albumin (BSA) to each well followed by an overnight incubation at 4°C. After being rinsed with PBS containing 0.05% Tween-20 to excess virus, the wells were incubated with chicken antisera against A/goose/Guangdong/01/1996 (H5N1) virus for 5 h at 4°C. The wells were then extensive washed and subsequently incubated with HRP-linked goat-anti-chicken antibody (Sigma-Aldrich, www.sigmaaldrich.com) for 2 h at 4°C. The wells were washed again with PBS containing 0.05% Tween-20, and incubated with O-phenylenediamine in substrate solution containing 0.01% H_2_O_2_ for 10 min at room temperature. The reaction was stopped by adding 50 µl of 1M H_2_SO_4_ and the absorbance was determined at 492 nm.

### Site-directed mutagenesis and virus generation

The HA mutants of DKGX/35 and BHGQH/3 viruses were generated using reverse genetics as previously described [13]. A site-directed mutagenesis kit (Invitrogen, www.invitrogen.com) was used to create specific mutations in the HA gene by using the primers shown in Table 4. The plasmids used for transfection were prepared by using the QIAfilter™ Plasmid Midi kit (QIAGEN, www.qiagen.com). All the constructs were completely sequenced to ensure the absence of unwanted mutations.

### Deglycosylation using PNGase F

Deglycosylation was achieved by using PNGase F (New England Biolabs, www.neb.com). Virus was concentrated by ultrafiltration with Microcon YM-100 tubes (Millipore, www.millipore.com) and purified by using column chromatography with Sepharose 4FF (GE Healthcare, www.gehealthcare.com). The virus concentrates were denatured according to manufacturer's instructions, and then deglycosylated by incubation at 37°C for 16 h with the PNGase F enzyme in the buffer provided by the manufacturer and NP40 at a final concentration of 1% (provided with the enzyme). Deglycosylated samples were analyzed by SDS-PAGE and western blot.

### Western blot analysis

Virus samples were analyzed by SDS-PAGE and Western immunoblotting as described previously [51]. Chicken antisera induced by the pCAGG-HA DNA vaccine [52] was used as the primary antibody, and IRDye™700DX-conjugated goat anti-chicken immunoglobulin G (IgG) (Rockland, www.rockland-inc.com) was used as the secondary antibody.

### Detection of α-2,3 glycans and α-2,6 glycans in guinea pig respiratory tissues

Paraffin-embedded, surgically removed, normal guinea pig upper-to-lower respiratory tract tissues were cut into 5 µm thick sections with a microtome. The sections were mounted on 3-aminopropyltrethoxy-silane (APS)-coated slides (Matsunami Glass Ind., Ltd., www.matsunami-glass.co.jp), deparaffinized in xylene, and rehydrated with alcohol. To detect sialyloligosaccharides reactive with α-2,3 glycan- or α-2,3 glycan-specific lectins, the sections were incubated with 250 µl of FITC-labeled *Sambucus nigra* (SNA) lectin (Vector Laboratories, www.vectorlabs.com) or biotinylated *Maackia amurensis* (MAA) lectin (Vector Laboratories, www.vectorlabs.com) overnight at 4°C. After three washes with Tris-buffered saline (TBS, pH 7.6), the sections were incubated with Alexa Fluor 594-conjugated streptavidin, (Molecular Probes, Inc., www.invitrogen.com) for 2 h at room temperature. They were then counterstained with 4′,6-diamino-2-phenylindole, dihydrochloride (DAPI; Dojindo Molecular Technologies, Inc., www.dojindo.com). After three washes with TBS, the sections were then mounted on cover glasses and observed with a fluorescence microscope (ECLIPSE TE300 with a fluorescence equipment mercury set, Nikon Co., www.nikonusa.com). Photographs were taken with a digital microscope camera (Olympus DP70, Olympus Optical Co., Ltd., www.olympus.com).
